# Hospital Chaplains to the Rescue

**Published:** 1920-05-22

**Authors:** 


### Hospital Chaplains to the Rescue.

THE report of the Commission upon the city churches, which recommends, with certain safeguards, the eventual demolition of nineteen city churches, mostly the work of Christopher Wren, has caused, and rightly caused, immense disquiet. Though the admirable Society for the Protection of Ancient Buildings is organising opposition to a policy which, from its own point of view, cannot be regarded as other than one of vandalism, the question is far more than a merely architectural one. Hospital chaplains and all the clergy concerned to support the Hospital Sunday Fund should be alive to the nature of the proposal. It is defended upon the ground that the eventual sale of the sites now occupied by the choicest of London's modern architectural heritage would produce a revenue of £100,000 a year, remove the "scandal" of rich city livings, to which little or no parish work is attached, and help to fill the church's admittedly impoverished coffers. By this means, the defenders of the proposal argue, poor benefices might be financially strengthened, new livings endowed, and so forth. Alive to the importance of these arguments, we must yet point out that the defence of the policy is, if possible, worse than the policy itself. For, architectural considerations apart, the defence of the well-endowed city living with little or no parochial work is a vital matter. These livings, in the first place, supply pensions not otherwise satisfactorily available for the elder clergy whose active parish work is mostly done; and, which is far more important, they serve to maintain those clergy who are primarily thinkers and scholars, who exist to keep the fountain of traditional theology pure and fresh, and give in the old phrase, that *otium cum dignitate*, without which the scholar, the thinker, and the mystic cannot do the work for which God made them. Lord Phillimore's letter in *The Times* of May 14 raises, rather than disposes of, this vital point.

After the somnolence and scepticism which descended upon the Church in the eighteenth century, the Wesleyans on one hand, the Tractarians on the other, arose to awaken England's religious life. These movements, fruitful as they became, were yet partial. To the mass of the clergy the appeal of that which became known as "muscular Christianity" was more strong, because everyone, even the least capable, was able to understand it. The weakness of "muscular Christianity" was a tendency to differentiate the clergy from the layman only by "the cloth" they wore, and to substitute "C.O.S. work" for faith and conviction. That tendency is with us still, and the policy of the Commission upon the city churches is the latest expression of it. But, just as the claims of Hospital Sunday can never be adequately presented without a lively personal faith in the gospel of personal service in the days of health for the cause of the sick, so the larger faith of the clergy and a standard of thought cannot be maintained in their purity, unless room is found in the "many mansions" of the Church for those to whom parochial work is not the be-all and the end-all of the Christian ministry. For these reasons we hope that hospital chaplains and all the clergy who believe in Hospital Sunday will work to defeat a policy which is really suicidal to the Church. Cannot the Bishop of London and Dean Inge give them an inspiring lead?

